# Genomic prediction with non-additive effects in beef cattle: stability of variance component and genetic effect estimates against population size

**DOI:** 10.1186/s12864-021-07792-y

**Published:** 2021-07-07

**Authors:** Akio Onogi, Toshio Watanabe, Atsushi Ogino, Kazuhito Kurogi, Kenji Togashi

**Affiliations:** 1 Department of Plant Life Science, Faculty of Agriculture, Ryukoku University, 1-5, Yokotani, Seta, Oe-cho, Shiga 520-2194 Otsu, Japan; 2 Maebashi Institute of Animal Science, Livestock Improvement Association of Japan, Inc, 371-0121 Maebashi, Japan; 3 Cattle Breeding Department, Livestock Improvement Association of Japan, Inc, 135-0041 Tokyo, Japan

**Keywords:** Epistasis, Dominance, Genomic selection, Mixed model, GBLUP

## Abstract

**Background:**

Genomic prediction is now an essential technology for genetic improvement in animal and plant breeding. Whereas emphasis has been placed on predicting the breeding values, the prediction of non-additive genetic effects has also been of interest. In this study, we assessed the potential of genomic prediction using non-additive effects for phenotypic prediction in Japanese Black, a beef cattle breed. In addition, we examined the stability of variance component and genetic effect estimates against population size by subsampling with different sample sizes.

**Results:**

Records of six carcass traits, namely, carcass weight, rib eye area, rib thickness, subcutaneous fat thickness, yield rate and beef marbling score, for 9850 animals were used for analyses. As the non-additive genetic effects, dominance, additive-by-additive, additive-by-dominance and dominance-by-dominance effects were considered. The covariance structures of these genetic effects were defined using genome-wide SNPs. Using single-trait animal models with different combinations of genetic effects, it was found that 12.6–19.5 % of phenotypic variance were occupied by the additive-by-additive variance, whereas little dominance variance was observed. In cross-validation, adding the additive-by-additive effects had little influence on predictive accuracy and bias. Subsampling analyses showed that estimation of the additive-by-additive effects was highly variable when phenotypes were not available. On the other hand, the estimates of the additive-by-additive variance components were less affected by reduction of the population size.

**Conclusions:**

The six carcass traits of Japanese Black cattle showed moderate or relatively high levels of additive-by-additive variance components, although incorporating the additive-by-additive effects did not improve the predictive accuracy. Subsampling analysis suggested that estimation of the additive-by-additive effects was highly reliant on the phenotypic values of the animals to be estimated, as supported by low off-diagonal values of the relationship matrix. On the other hand, estimates of the additive-by-additive variance components were relatively stable against reduction of the population size compared with the estimates of the corresponding genetic effects.

**Supplementary Information:**

The online version contains supplementary material available at 10.1186/s12864-021-07792-y.

## Background

Genomic prediction is a technology using whole-genome information to predict the genetic merits of genotypes [1]. Genomic selection, selection based on genomic prediction, was first implemented in dairy cattle and has been shown to increase genetic gain more quickly than the conventional method based only on pedigree records and phenotypic information [2]. Now, genomic prediction/selection is recognised as a useful tool for improving quantitative traits. Because genetic ability transmittable to the progeny is important in animal breeding, genomic prediction usually focuses on prediction of breeding values. Nevertheless, prediction of the non-additive genetic effects (dominance and epistatic effects) has also been of interest for, for example, predicting future performance in farms, designing mating or increasing the accuracy of predicting the additive components [3]. Prediction of the non-additive effects can be achieved by explicitly adding the corresponding effects to the prediction models or by using non-linear prediction methods such as neural networks [4] and reproducing kernel Hilbert space regression with non-linear kernels [5]. A merit of the former explicit approach is that breeding values can be predicted, which is more informative for breeding purposes.

The contributions of the non-additive effects to the prediction of phenotypes appear to be case-dependent. For example, dominance effects slightly increased accuracy in several pig studies [6, 7], whereas the effects achieved little improvement in dairy [8] and beef [9] cattle populations, although plenty of dominance variance was found for several traits in the beef cattle study [9]. Epistasis effects improved phenotype prediction in *Drosophila* [10], whereas no improvement was reported in the aforementioned pig study [6], regardless of the relatively large proportion of additive-by-additive variance. The improvement by adding non-additive effects is thus elusive, and empirical evaluation of the populations and traits of interest is necessary.

Japanese Black is a major beef cattle breed unique to Japan, which is now known as Wagyu and associated with an abundance of fat and flavour. Genomic prediction of the breed was assessed using the single-step approach, which combines SNP information with pedigree information in a single genetic relationship matrix [11]. Now, genomic estimated breeding values (breeding values from genomic prediction) are used for sire evaluation. The purpose of this study is to assess the possibility of using non-additive genetic effects for predicting cattle phenotypes in farms for Japanese Black cattle. First, we estimated the non-additive genetic variance components for carcass traits using 9850 animals. Then, we examined the accuracy of predicting phenotypes using both additive and non-additive effects with cross-validation. Finally, to assess the stability of the variance component and genetic effect estimates against the population size, we conducted subsampling analysis with different sampling sizes.

## Results and discussion

To assess the possibility of using non-additive genetic effects for predicting cattle phenotypes in farms for Japanese Black cattle, we estimated the non-additive genetic variance components for six carcass traits using 9850 animals. The traits analysed were carcass weight (CW, kg), rib eye area (REA, cm^2^), rib thickness (RT, cm), subcutaneous fat thickness (SFT, cm), yield rate (YI, %) and beef marbling score (BMS). A summary of the phenotypic values is presented in Table 1. Total six single-trait animal models with different combinations of genetic effects, Models A, D, AA, AA2, AD, and Full, were fitted for each trait (Methods).

**Table 1:** Mean (SD) values of phenotypes

	CW (kg)	REA (cm^2^)	RT (cm)	SFT (cm)	YI (%)	BMS (1–12)
Heifer	443.7 (52.5)	58.5 (9.7)	7.92 (0.89)	2.89 (0.81)	74.2 (1.6)	6.6 (2.0)
Steer	489.4 (54.3)	60.8 (10.6)	8.03 (0.85)	2.49 (0.71)	74.3 (1.6)	6.8 (2.1)

*CW* carcass weight, *REA* rib eye area, *RT* rib thickness, *SFT* subcutaneous fat thickness, *YI* yield rate, *BMS* beef marbling score

**Fig. 1:**
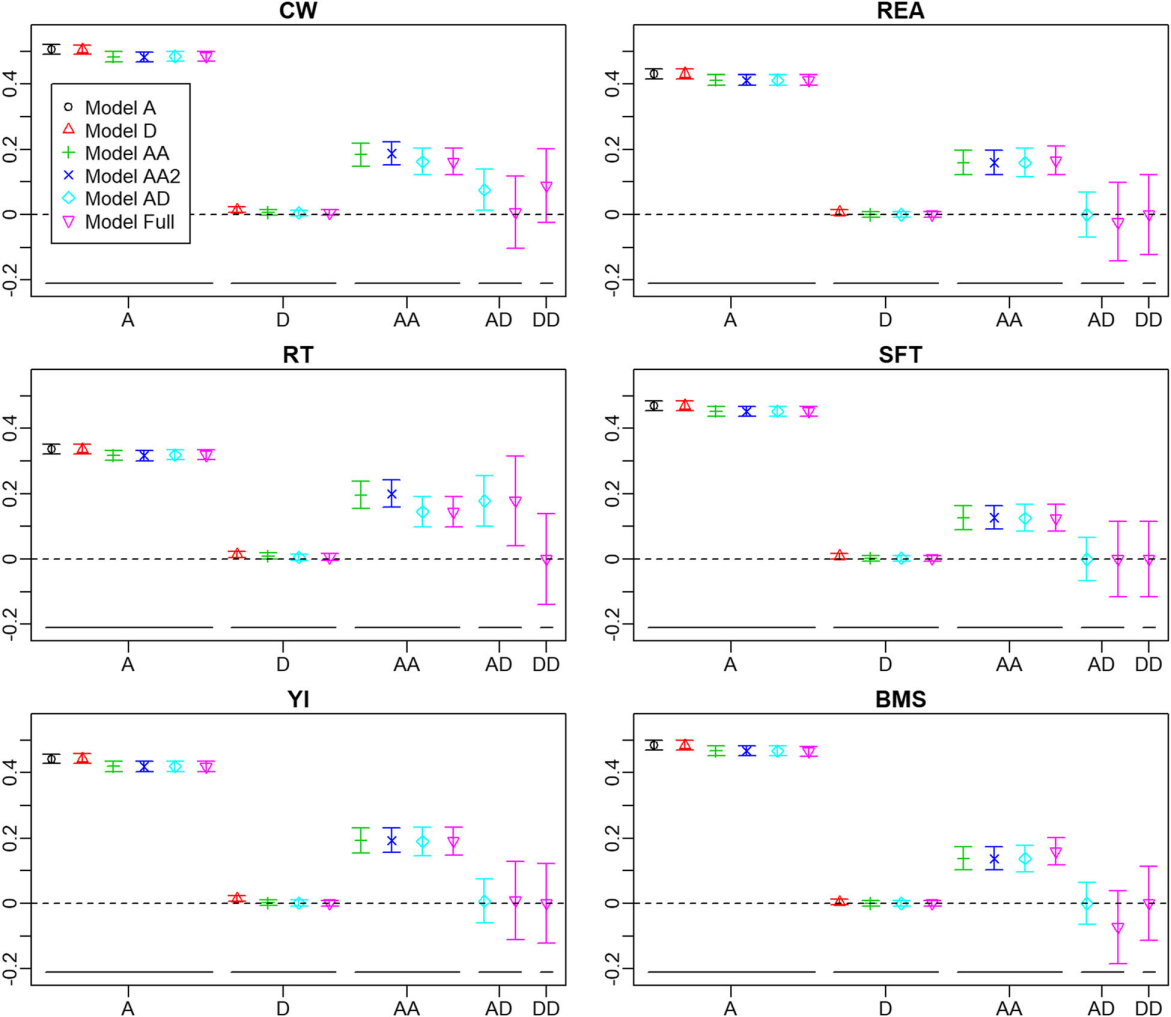
Proportions of each variance component relative to phenotypic variance. Different colours indicate different models. Bars are standard errors. A, additive variance; D, dominance variance; AA, additive-by-additive variance; AD, additive-by-dominance variance; dominance-by-dominance variance; CW, carcass weight; REA, rib eye area; RT, rib thickness; SFT, subcutaneous fat thickness; YI, yield rate; BMS, beef marbling score

The proportions of each variance component relative to the total phenotypic variance are shown in Fig. 1. The proportions of the additive variance component were 0.483–0.506 for CW, 0.412–0.431 for REA, 0.316–0.336 for RT, 0.452–0.470 for SFT, 0.419–0.443 for YI and 0.465–0.484 for BMS. The proportions of the additive variance slightly decreased when the additive-by-additive (AA) components were added. The proportions of the dominance variance were almost 0 for each trait. The proportions of the AA component were generally one-quarter to one-half of those of the additive component, and the estimates were stable across the models: 0.162–0.183 for CW, 0.160–0.166 for REA, 0.145–0.195 for RT, 0.126–0.126 for SFT, 0.190–0.192 for YI and 0.138–0.160 for BMS. The proportions of the additive-by-dominance (AD) variance and the dominance-by-dominance (DD) variance were accompanied by large standard errors and appeared to be negligible, except for AD of RT.

For the Japanese Black cattle, non-additive genetic variance components of carcass traits have not been reported previously, so this is the first report on them. For growth traits (birth weight, market weight and average daily gain), on the other hand, dominance variances were estimated for this breed using pedigree records [12]. The proportions of dominance variances relative to the phenotypic variances of these traits were 0.00 ± 0.00, 0.13 ± 0.06 and 0.09 ± 0.05, respectively. Although market weight would be highly correlated with CW, the proportion for market weight was higher than that for CW in our study (0.005–0.015 depending on the model). This would be attributable to the difference in the studied populations. In the preceding study, the population consisted of animals dispersed across multiple small islands where different rearing and breeding systems were used. Thus, the population might be genetically diverse, which could result in greater dominance variances. On the other hand, the genetic structure of the population in the present study was more uniform, as illustrated by the population structure analysis (Figure S1), and consequently lower estimates might be obtained. Dominance variances for carcass traits can be found in other breeds, for example, CW of Simmental (the proportion relative to the phenotypic variance was 0.158) [13] and intramuscular fat and carcass retail yield of a population consisting of multiple breeds (0.10 ± 0.03 and 0.18 ± 0.06, respectively) [9]. Dominance variances of growth traits were also reported in other breeds of beef cattle, for example, for yearling weight of Brahman and Tropical composite (up to 0.13 and 0.10, respectively) [14], 205-day body weight in synthetic populations (0.00–0.52) [15] and post-weaning gain for Limousin (0.099 ± 0.016) [16]. Regardless of the traits and populations, zero or near zero dominance variances as observed in our study appear to be rare.

Our study showed that AA variance constituted 0.126–0.195 of the phenotypic variance of carcass traits (Fig. 1). The epistatic genetic variance in beef cattle was reported in some of the above-mentioned studies; for yearling weight of Brahman and Tropical composite, the proportions of the AA component relative to the phenotypic variance were approximately 0.20 and 0.25, respectively [14], and for 205-day body weight in synthetic populations, the proportions of AA were almost negligible, at 0.000–0.003 [15]. Besides beef cattle, the AA components were estimated as 0.093–0.098 [6] for pig daily gain and as typically 0.05–0.20 for growth traits of tilapia [17]. Although the detected amount of epistatic variance would vary depending on the trait and population, the AA variance components estimated in our study appear to be moderate or relatively high. A recent study illustrates that models with epistatic effects can show higher prediction accuracy than additive models when the linkage disequilibrium (LD) between markers is low [18]. Thus, it is suggested that epistatic variance components can be overestimated in such situation. The *r*^*2*^ values between contiguous SNPs in our study are 0.126, 0.293, and 0.771 at 50 %, 70 %, and 90 % quantiles, respectively. These values largely correspond with the Arabidopsis data with 10^5^ and 10^6^ markers in Ref. [18] where the superiority of the epistasis models over the additive models in prediction accuracy disappeared. Moreover, to verify this, we estimated the variance components with model AA2 using **AA** generated from the reduced numbers of SNPs; the SNP densities were reduced to 0.125-, 0.25-, 0.5- and 0.75-fold of the original density by sampling the corresponding number of SNPs from the consecutive eight SNPs. As a result, the AA variance increased with the reduction in the density (Figure S2). However, the variance at 0.75-fold was almost equal to that at the original density for all traits. Thus, we conclude that the LD issue would not affect the estimates of the AA components in our study.

Based on the results of variance component estimation, for the cross-validation (CV) of traits except for RT, we adopted Model AA2, in which the dominance effect from Model AA was removed. For RT, we adopted Model AD because of its relatively large AD component. Although Akaike information criterion (AIC) tended to support complex models such as Model Full and Model AD (Table S1), our choice would be more reasonable. Actually, the predictive accuracy of the models with the lowest AICs were almost equivalent to those of the models shortlisted in this study (Table S2). The estimates of variance components with the selected models are presented in Table 2. In Table 3, directional dominance estimated as the slope of the proportion (%) of heterozygotes on phenotypic values with the selected models is shown. Dominance and dominance-related epistatic variances (AD and DD) were negligible for most traits, whereas directional dominance was not negligible for CW, REA, RT and SFT, and almost zero for the others. Inbreeding depression can occur for the traits with positive slopes, so care is needed not to increase the inbreeding coefficients.

**Table 2:** Variance component estimates (standard errors)

	CW	REA	RT	SFT	YI	BMS
A	1061.2 (50.9)	37.2 (2.0)	0.200 (0.012)	0.236 (0.012)	0.959 (0.050)	1.754 (0.086)
D			0.004 (0.006)			
AA	412.5 (78.0)	14.4 (3.4)	0.091 (0.030)	0.066 (0.018)	0.443 (0.087)	0.517 (0.132)
AD			0.112 (0.049)			
R	724.2 (70.8)	38.6 (3.1)	0.222 (0.042)	0.219 (0.017)	0.888 (0.079)	1.488 (0.122)

*CW *carcass weight, *REA *rib eye area, *RT* rib thickness, *SFT *subcutaneous fat thickness, *YI* yield rate, *BMS* beef marbling score, *A* additive, *D* dominance, *AA* additive-by-additive, *AD *additive-by-dominance, *R* residual

**Table 3:** Directional dominance (standard errors) estimated as the effect of the proportion (%) of heterozygotes on phenotypic values

CW	REA	RT	SFT	YI	BMS
4.255 (0.372)	0.366 (0.077)	0.052 (0.007)	0.041 (0.006)	−0.007 (0.012)	−0.017 (0.015)

*CW* carcass weight, *REA* rib eye area, *RT *rib thickness, *SFT *subcutaneous fat thickness, *YI* yield rate, *BMS* beef marbling score

**Fig. 2:**
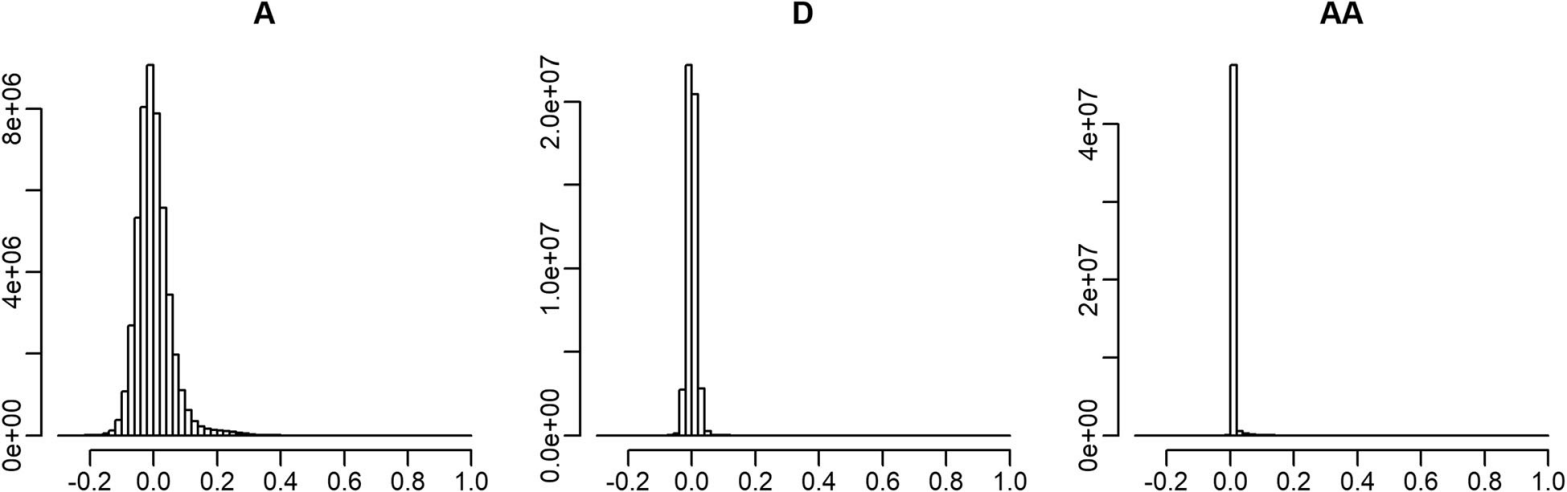
Comparison of off-diagonal elements among the realised relationship matrices, **A**, **D** and **AA**. **A**, additive matrix; **D**, dominance matrix; **AA**, additive-by-additive matrix

Predictive accuracy of the models was measured as the correlation coefficients between the phenotypes adjusted for fixed effects, and total genotypic values predicted in the CV. The predictive accuracy with Model A was moderate (0.449–0.630) and models with non-additive genetic effects (Model AD for RT and Model AA2 for the others) showed little gain of accuracy. The bias of prediction was also similar between the models (Table 4). We also evaluated the models in terms of predictive accuracy and bias of additive genetic effects by comparing the predicted additive genetic effects with the adjusted phenotypic values. However, only slight difference was observed between the models (Table S3). In short, although certain proportions of the AA variance components were estimated, the effects had little influence on prediction. Similar results were reported in a study of pig; inclusion of the AA effects did not improve the predictive accuracy, despite the relatively large proportion of the variance component (0.093–0.098) [6]. It was also reported that, although the dominance variance was smaller than the AA (the proportion was 0.056), the dominance effects could increase the predictive accuracy. The small contribution of epistatic effects to prediction would result from the low off-diagonals of the relationship matrices. The off-diagonal elements of **AA** were much closer to zero than those of **A** and **D** because of the Hadamard product of **A** (Fig. 2). Roughly speaking, **AA** considers the proportions of genotype combinations shared between animals, but these proportions are expected to be very low. Thus, estimation of the AA effect of an animal will rely heavily on its phenotypic value, which makes prediction of this effect difficult.

**Table 4:** Predictive accuracy and bias in cross-validation

		CW	REA	RT	SFT	YI	BMS
Accuracy^a^	Additive^c^ model	0.630	0.541	0.449	0.550	0.546	0.596
	Non-additive^d^ model	0.632	0.546	0.450	0.552	0.552	0.599
Bias^b^	Additive model	0.399	0.290	0.201	0.302	0.295	0.352
	Non-additive model	0.401	0.294	0.203	0.303	0.299	0.354

*CW* carcass weight, *REA* rib eye area, *RT* rib thickness, *SFT* subcutaneous fat thickness, *YI* yield rate, *BMS* beef marbling score

^a^Pearson’s correlation coefficient between adjusted phenotypic values and predicted total genotypic values

^b^Coefficient obtained by regressing predicted total genotypic values on adjusted phenotypic values

^c^Model A

^d^Model AD for RT and Model AA2 for the other traits

**Fig. 3:**
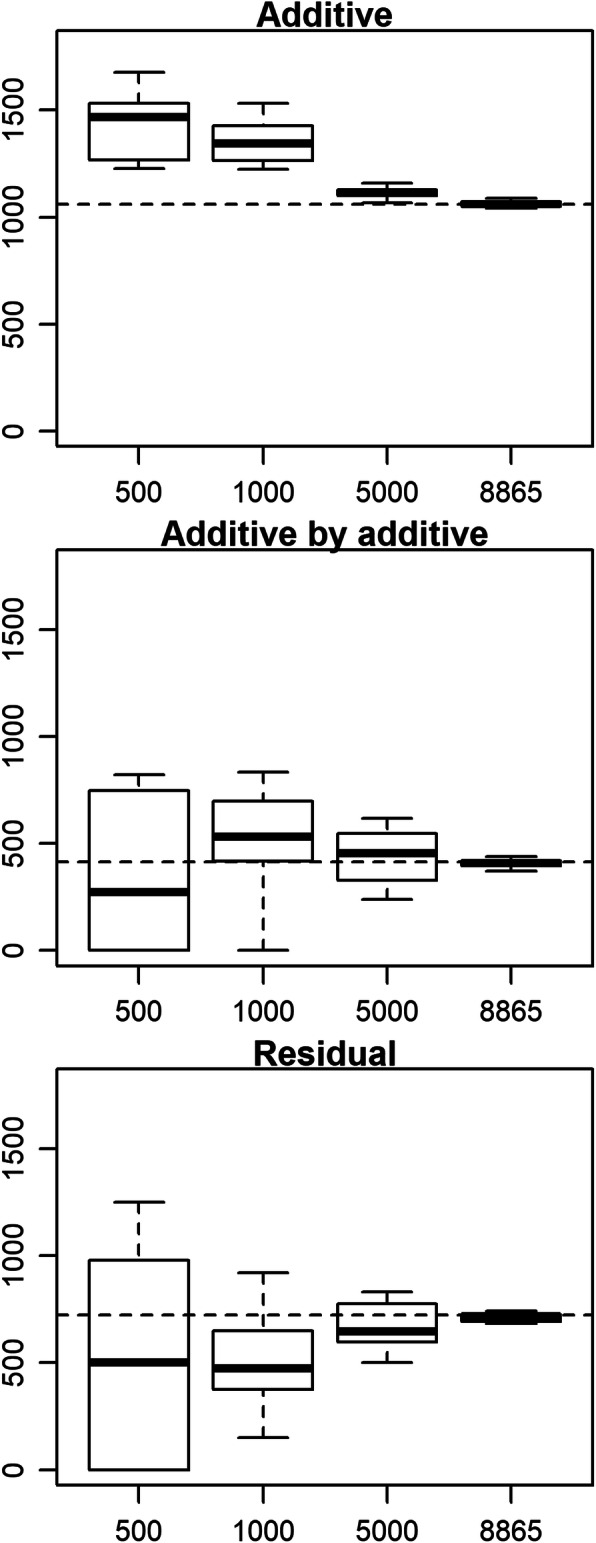
Variance component estimates when population size was reduced by subsampling. The x-axis indicates the population size. The phenotypes of carcass weight adjusted with the fixed effects estimated from the full data with Model AA2 were used. The horizontal broken lines indicate the estimates from the full data with Model AA2

**Fig. 4:**
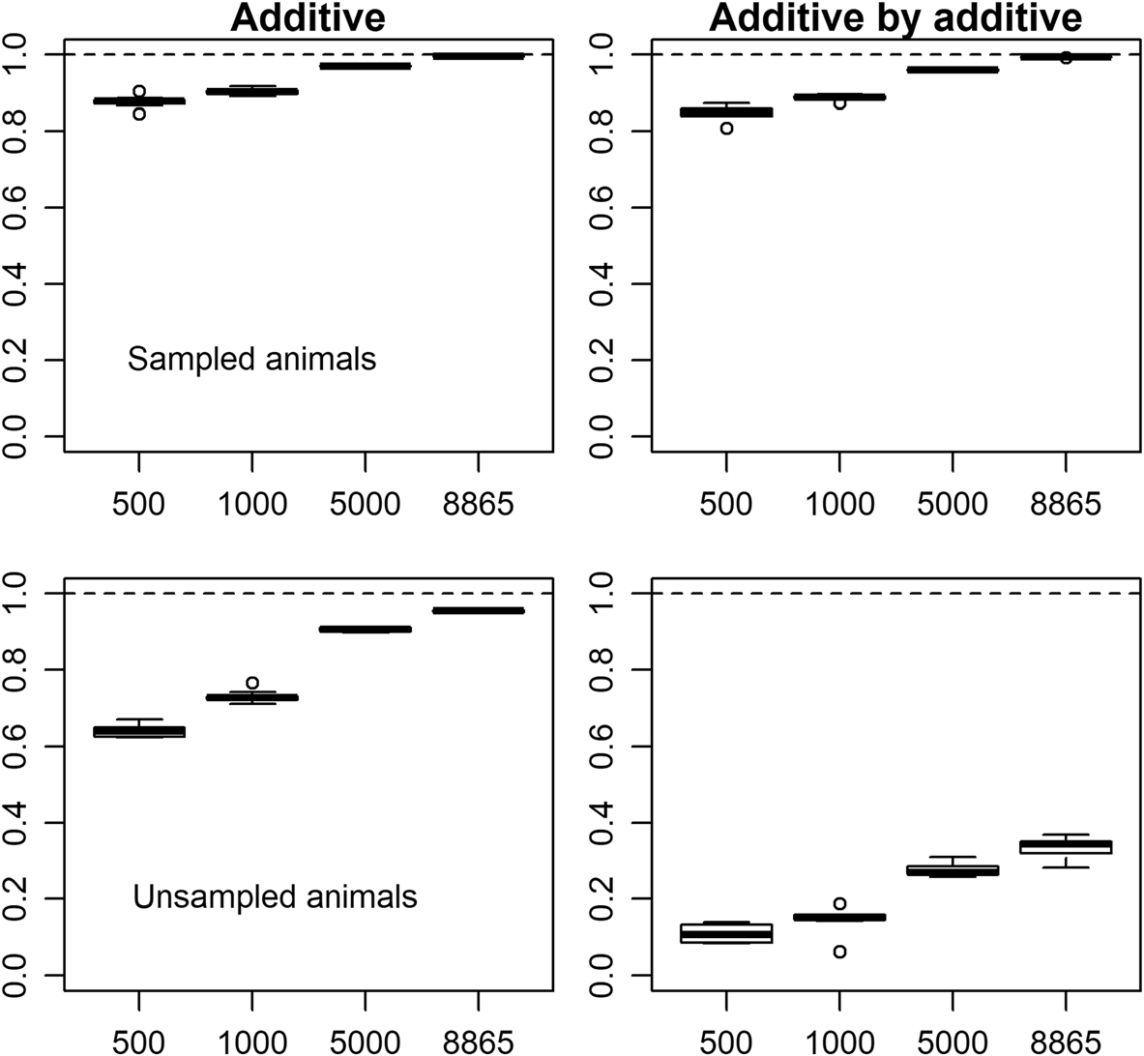
Correlations between the genetic effects estimated from the subsampled data sets and the full data set. The x-axis indicates the population size and the y-axis indicates the correlation coefficient. The left panels are the additive effects and the right panels are the additive-by-additive effects. The upper panels are correlations for the sampled animals (i.e. animals with phenotypes) and the lower panels are correlations for the unsampled animals (i.e. animals without phenotypes). The phenotypes of carcass weight adjusted with the fixed effects estimated from the full data with Model AA2 were used. The broken lines are drawn at *y* = 1.0

When the population size was reduced by subsampling, the estimates of the additive variance tended to be inflated as the size decreased (Fig. 3). The estimates of the AA and residual variances were highly unstable when the sizes were 500 and 1000, and gradually converged to the estimates from the full data as the size expanded. These results suggest that (1) the variance component estimates of the epistatic effects are unreliable when the population size is quite small (here, < 1000), but (2) the estimates are stable against the population size (or choice of animals) once the population reaches a certain size (> 5000). Thus, the AA variance components estimated from the full data would be reliable. Different tendencies were observed for the genetic effects (Fig. 4). For the genetic effects of sampled animals, regardless of the effect types (additive and AA), correlations between the estimates from the subsamples and the full data sets approached 1 as the population size increased (Fig. 4). However, for the genetic effects of unsampled animals, the correlations were quite different between the additive and AA effects. For the latter, *r* values were still 0.281–0.369 when 8865 animals were used (Fig. 4), although the estimated variance components at that sample size were almost equivalent to those from the full data (Fig. 3). These results clearly showed that estimation of the AA effects relied on the phenotypic values of the animals to be estimated and thus, prediction, or extrapolation, of the AA effects is difficult, as suggested by the comparison of the off-diagonal elements between the relationship matrices (Fig. 2). Considering that the population used in this study consists of paternal half-sib families, prediction of the AA effect (or epistatic effects) would be difficult unless more closely related counterparts (e.g. full-sibs or progeny) exist in the training population and/or an extremely large training population is available.

## Conclusions

The six carcass traits of Japanese Beef cattle showed moderate or relatively high levels of the AA variance components. However, incorporating the AA effects did not improve the predictive accuracy. Subsampling analysis revealed that estimation of the AA effects was highly reliant on the phenotypic values of the animals to be estimated. On the other hand, the estimates of the AA variance components were less affected by the choice or reduction in number of the phenotyped animals. This study illustrated this interesting contrast between the genetic effect and variance component estimation of epistatic effects.

## Methods

### Animals and traits

The data used in this study were collected in the progeny testing programme conducted by the Livestock Improvement Association of Japan, Inc. (LIAJ). The number of animals with phenotypes was 9850, of which 4142 were heifers and 5708 were steers. These animals had been fattened in 65 farms, of which two were experimental stations of LIAJ, one was a national experimental station, and the rest were commercial farms. Slaughter dates ranged from April 2012 to December 2018, and the mean ± SD ages at slaughter were 30.1 ± 1.5 (heifer) and 28.8 ± 1.2 months (steer). These animals were the offspring of a total of 487 sires. The number of offspring per sire was a mean of 20.2 ± 42.3, ranging from 1 to 554.

All traits (CW, REA, RT, SFT, YI, and BMS) were measured by the Japan Meat Grading Association, which is an official grader in Japan. The value of BMS was an integer between 1 and 12, with higher values indicating more abundant intramuscular fat. YI was estimated from CW, REA, RT and SFT by the grader.

### SNP genotypes

The animals with phenotypes were genotyped for genome-wide SNPs using Illumina BovineLD Genotyping BeadChip ver. 1, 1.1 or 2 (Illumina, CA, USA). The call rate of these animals was 0.998 ± 0.003. Using Beagle ver. 4.0 [19], the BovineLD genotypes were expanded to the SNP genotypes of the Illumina BovineSNP50 BeadChips ver. 2, with the BovineSNP50 genotypes of 1223 sires and 4 dams as a reference. After pruning with a minor allele frequency threshold of > 0.05, 33,738 SNPs were used in this study.

### Genetic relationship matrix

The realised genetic relationship matrices for the additive and dominance effects (**A** and **D**, respectively) were calculated using the imputed SNP genotypes following the natural and orthogonal interactions model [20, 21]. In addition, AA, AD and DD relationship matrices (**AA**, **AD** and **DD**, respectively) were calculated as the Hadamard products between the corresponding matrices [21]. The diagonals of these matrices were scaled such that the trace of the matrix (i.e. sum of the diagonal elements) was equal to the number of animals [21]. As implied from a study by Vitezica et al. [21] and as illustrated by Jiang et al. [22], the Hadamard product between the corresponding matrices includes the squares of the same SNP and reciprocal products between two SNPs (e.g., SNP A by SNP B and SNP B by SNP A). Thus, for the AA interaction, which was shown to have non-negligible variance components for all traits, we calculated **AA** avoiding these issues and compared it with **AA** calculated with the Hadamard product of **A**. However, the off-diagonal elements were almost the same between the two matrices (the Pearson correlation coefficient > 0.999), thus, we used the **AA** generated by the Hadamard product.

### Statistical models

Single-trait animal models implemented in the R package sommer v4.09 and v4.12 [23] were used for variance component estimation and phenotype prediction. The base model includes the additive genetic effect as a random effect. The model is referred to as Model A, and can be written as:

Model A $$\mathbf{Y}=\mathbf{X}\mathbf{B}+{\mathbf{U}}_{\mathbf{A}}+\mathbf{E}$$

where **Y** is the phenotypes, **X** is the incidence matrix of fixed effects, **B** is the fixed effects, **U**_**A**_ is the additive genetic effect and **E** is the residuals. Here the additive genetic effect refers to the effect of least-squares meanings [21]. The fixed effects included sex, farm, slaughter date (month-year combinations), age in months and the proportion of heterozygotes of SNP genotypes. This last effect was included to consider the directional effects of dominance [24]. The average heterozygosity was 0.318 ± 0.016. Age in months and the proportion of heterozygotes were standardised before model fitting. Farms with few animals (typically < 10) were grouped together according to geographical regions, resulting in 22 levels. **U**_**A**_ and **E** were assumed to follow ${\mathbf U}_{\mathbf A}\sim\mathrm{MVN}\left({\mathbf 0},\;\;\;{\mathbf A}\sigma_A^2\;\right)$, and $\mathbf E\sim\mathrm{MVN}\left({\mathbf 0},\;\;\;{\mathbf I}\sigma_E^2\right)$ respectively, where MVN denotes the multivariate normal distributions, ${\sigma }_{A}^{2}$ and ${\sigma }_{E}^{2}$ are the variance components, and **I** is the identity matrix. Model D includes the dominance component as:

Model D $$\mathbf{Y}=\mathbf{X}\mathbf{B}+{\mathbf{U}}_{\mathbf{A}}+{\mathbf{U}}_{\mathbf{D}}+\mathbf{E}$$

where $${\mathbf U}_{\mathbf D}\sim\mathrm{MVN}\left({\mathbf 0},\;\;\;\mathrm {\mathbf D}\sigma_D^2\right)$$ .

The AA, AD and DD variance components were estimated by adding the corresponding terms to Model D incrementally, that is:

$$\mathbf{Y}=\mathbf{X}\mathbf{B}+{\mathbf{U}}_{\mathbf{A}}+{\mathbf{U}}_{\mathbf{D}}+{\mathbf{U}}_{\mathbf{A}\mathbf{A}}+\mathbf{E}$$ (Model AA),

$\mathbf{Y}=\mathbf{X}\mathbf{B}+{\mathbf{U}}_{\mathbf{A}}+{\mathbf{U}}_{\mathbf{D}}+{\mathbf{U}}_{\mathbf{A}\mathbf{A}}+{\mathbf{U}}_{\mathbf{A}\mathbf{D}}+\mathbf{E}$ (Model AD) and

$$\mathbf{Y}=\mathbf{X}\mathbf{B}+{\mathbf{U}}_{\mathbf{A}}+{\mathbf{U}}_{\mathbf{D}}+{\mathbf{U}}_{\mathbf{A}\mathbf{A}}+{\mathbf{U}}_{\mathbf{A}\mathbf{D}}+{\mathbf{U}}_{\mathbf{D}\mathbf{D}}+\mathbf{E}$$ (Model Full),

respectively, where ${\mathbf U}_{\mathbf{AA}}\sim\mathrm{MVN}\left({\mathbf 0},\;\;\;\mathbf{AA}\sigma_{AA}^2\right)$, ${\mathbf U}_{\mathbf{AD}}\sim\mathrm{MVN}\left({\mathbf 0},\;\;\;\mathbf{AD}\sigma_{AD}^2\right)$ and ${\mathbf U}_{\mathbf{DD}}\sim\mathrm{MVN}\left({\mathbf 0},\;\;\;\mathbf{DD}\sigma_{DD}^2\right)$. After model fitting, the dominance component was found to be negligible for most traits. Thus, we also fitted the following model that does not include the dominance component:

$$\mathbf{Y}=\mathbf{X}\mathbf{B}+{\mathbf{U}}_{\mathbf{A}}+{\mathbf{U}}_{\mathbf{A}\mathbf{A}}+\mathbf{E}$$ (Model AA2).

### Cross-validation

The predictive ability of the models was examined by 10-fold CV. For each trait, one model with non-additive effects was empirically selected from Models D, AA, AA2, AD and Full based on the full data analyses, as described in the Results section. Because genetic population structure was negligible in this population (Figure S1), the animals with phenotypes were randomly split into 10 folds such that all levels of slaughter dates and farms were always included in the training populations. Predictive accuracy was evaluated using Pearson’s correlation coefficient (*r*) between the phenotypic values adjusted with the fixed effects estimated from the full data and the summation of predicted genetic effects (total genotypic values). The fixed effects used for this adjustment were estimated with the same non-additive models as prediction. Prediction bias was evaluated using the coefficient obtained by regressing the predicted genetic effects on the adjusted phenotypic values.

### Subsampling

To investigate the stability of variance component and genetic effect estimates against the population size, we reduced the population size by subsampling and fitted the models. The sizes considered were 500, 1000, 5000 and 8865. The last of these corresponds to the training population size of CV (9850 − 985). We used the phenotypes of CW adjusted with the fixed effects estimated with Model AA2 from the full data. For each population size, animals were randomly sampled without replacement. Then, the models that had the same random effects as Model AA2, but only had the intercept as the fixed effect, were fitted. The estimates of variance components and genetic effects (additive and AA effects) were compared with those from the full data. For the genetic effects, *r* was calculated between the estimates from the subsampled and full data sets. This calculation was performed for the sampled animals (i.e. animals with phenotypes) and unsampled animals (animals without phenotypes) separately. The latter corresponds to comparing the predicted genetic effects with the estimates from the full data.

## Supplementary Information

**Additional file 1: Figure S1.** Genetic population structure estimated with eigen decomposition of the additive relationship matrix. (A) Cumulative proportions of variances of principal components (PCs). (B) Plot for PC 1 and PC 2. (C) Plot for PC 3 and PC4. **Figure S2.** The additive-by-additive (AA) genetic variance estimated from reduced SNP densities. Model AA2 was used. The y-axes indicate the proportion of phenotypic variance explained by the AA variance. The x-axes indicate the SNP density represented as the proportion of the original density. Bars indicate the standard errors. The broken horizontal lines indicate the estimates at the original density. CW, carcass weight; REA, rib eye area; RT, rib thickness; SFT, subcutaneous fat thickness; YI, yield rate; BMS, beef marbling score. **Table S1.** Akaike information criteria (AIC). The best models are highlighted in bold. **Table S2.** Comparison of predictive accuracies between models with the lowest AIC and chosen models. **Table S3.** Predictive accuracy and bias of phenotypes using additive genetic effects in cross-validation.

## Data Availability

The phenotypic values adjusted for the non-additive models and the genetic relationship matrices (**A** and **D**) are available at Dryad (10.5061/dryad.tdz08kpz4).

## Abbreviations

CW: Carcass weight
REA: Rib eye area
RT: Rib thickness
SFT: Subcutaneous fat thickness
YI: Yield rate
BMS: Beef marbling score
AA: Additive-by-additive
AD: Additive-by-dominance
DD: Dominance-by-dominance
LD: Linkage disequilibrium
CV: Cross-validation
AIC: Akaike information criterion
LIAJ: The Livestock Improvement Association of Japan, Inc
