# Supplementary material for: Genomic prediction with non-additive effects in beef cattle: stability of variance component and genetic effect estimates against population size
Source: BMC Genomics. 2021 Jul 7;22:512. doi: 10.1186/s12864-021-07792-y (PMC8262069; doi:10.1186/s12864-021-07792-y)
Supplement: Supplementary file 1 — Additional file 1: Figure S1. Genetic population structure estimated with eigen decomposition of the additive relationship matrix. (A) Cumulative proportions of variances of principal components (PCs). (B) Plot for PC 1 and PC 2. (C) Plot for PC 3 and PC4. Figure S2. The additive-by-additive (AA) genetic variance estimated from reduced SNP densities. Model AA2 was used. The y-axes indicate the proportion of phenotypic variance explained by the AA variance. The x-axes indicate the SNP density represented as the proportion of the original density. Bars indicate the standard errors. The broken horizontal lines indicate the estimates at the original density. CW, carcass weight; REA, rib eye area; RT, rib thickness; SFT, subcutaneous fat thickness; YI, yield rate; BMS, beef marbling score. Table S1. Akaike information criteria (AIC). The best models are highlighted in bold. Table S2. Comparison of predictive accuracies between models with the lowest AIC and chosen models. Table S3. Predictive accuracy and bias of phenotypes using additive genetic effects in cross-validation. [file 12864_2021_7792_MOESM1_ESM.pptx]

## Slide 1
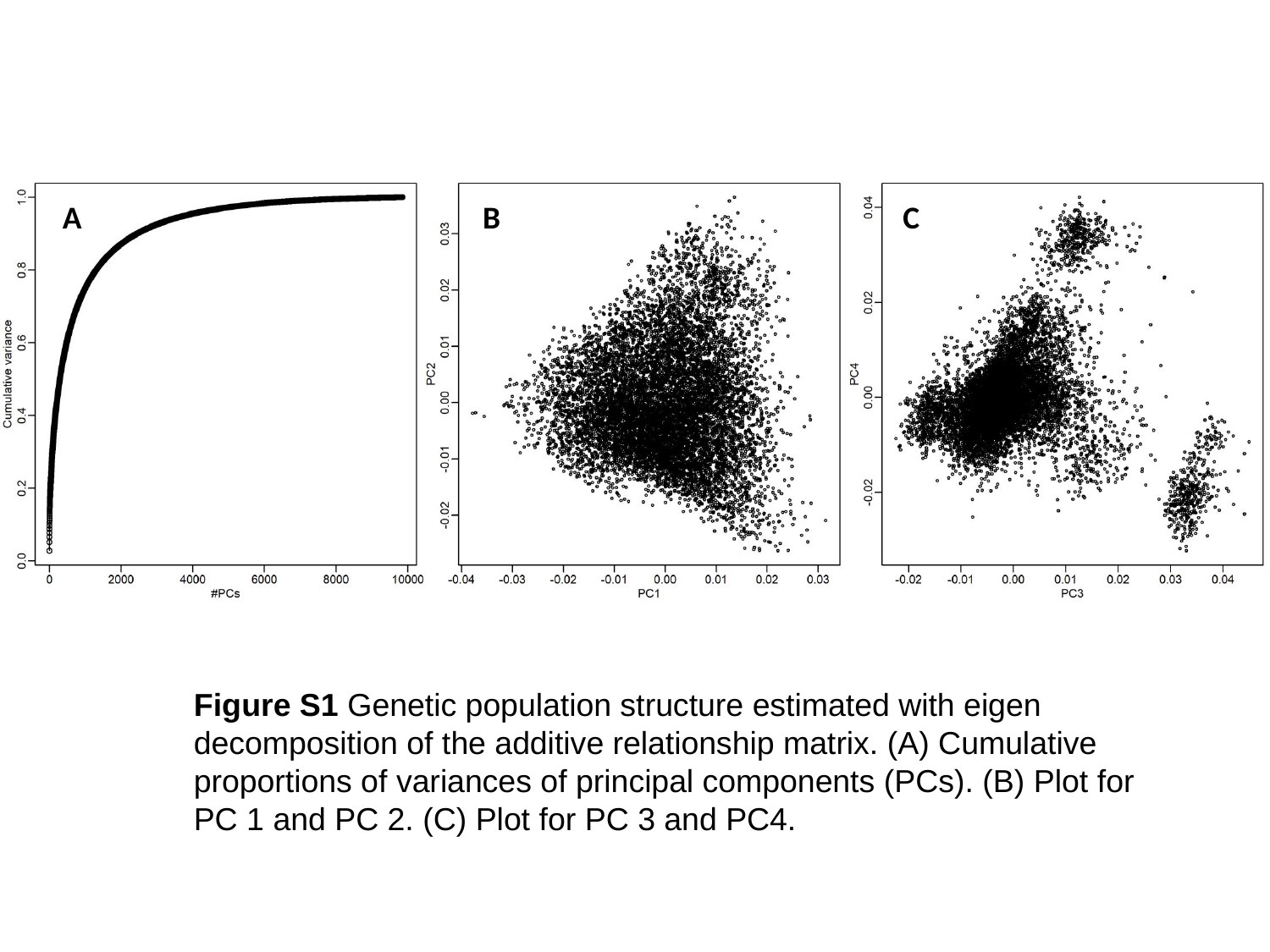

A
B
C
Figure S1 Genetic population structure estimated with eigen decomposition of the additive relationship matrix. (A) Cumulative proportions of variances of principal components (PCs). (B) Plot for PC 1 and PC 2. (C) Plot for PC 3 and PC4.

## Slide 2
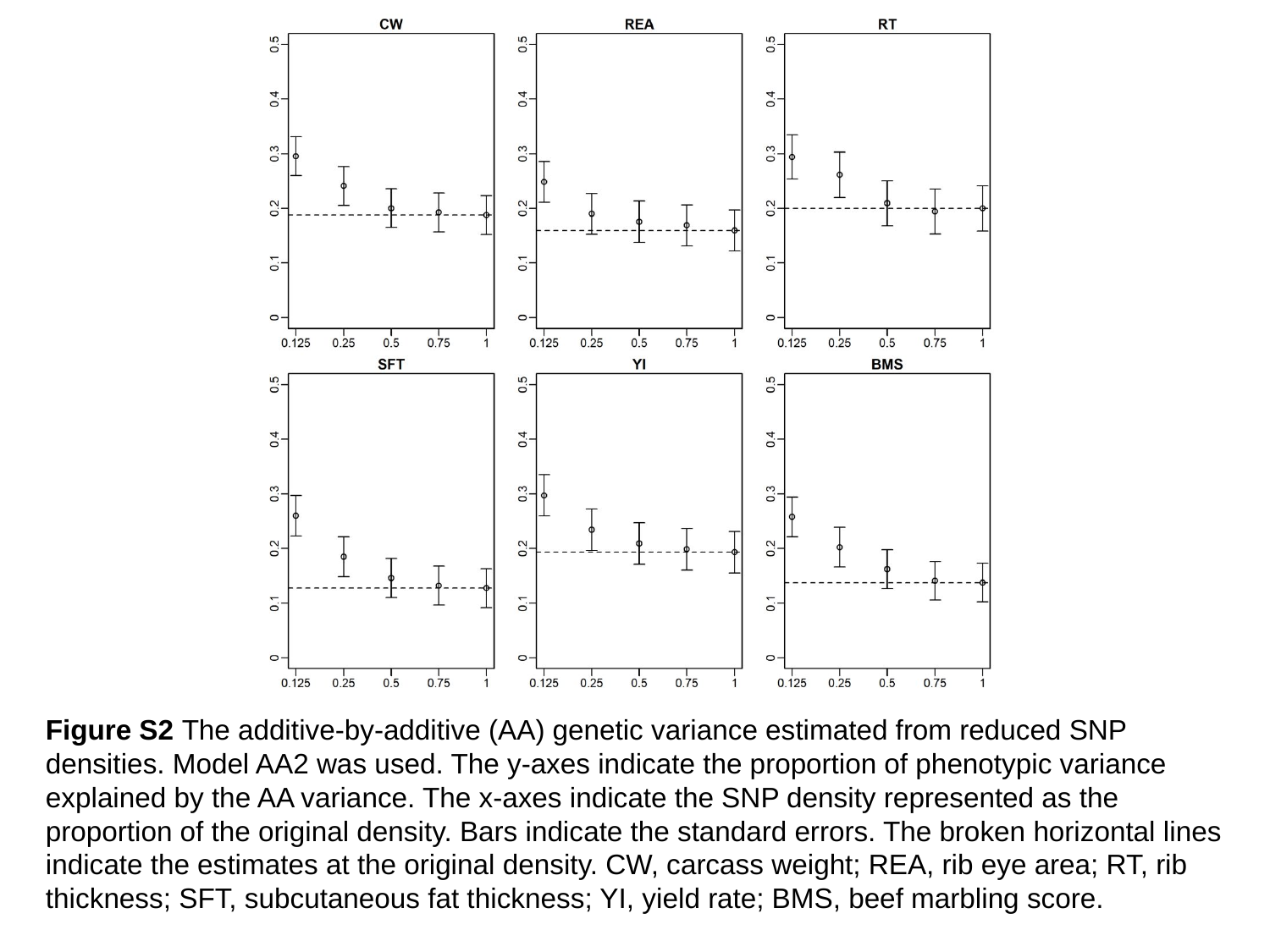

Figure S2 The additive-by-additive (AA) genetic variance estimated from reduced SNP densities. Model AA2 was used. The y-axes indicate the proportion of phenotypic variance explained by the AA variance. The x-axes indicate the SNP density represented as the proportion of the original density. Bars indicate the standard errors. The broken horizontal lines indicate the estimates at the original density. CW, carcass weight; REA, rib eye area; RT, rib thickness; SFT, subcutaneous fat thickness; YI, yield rate; BMS, beef marbling score.

## Slide 3
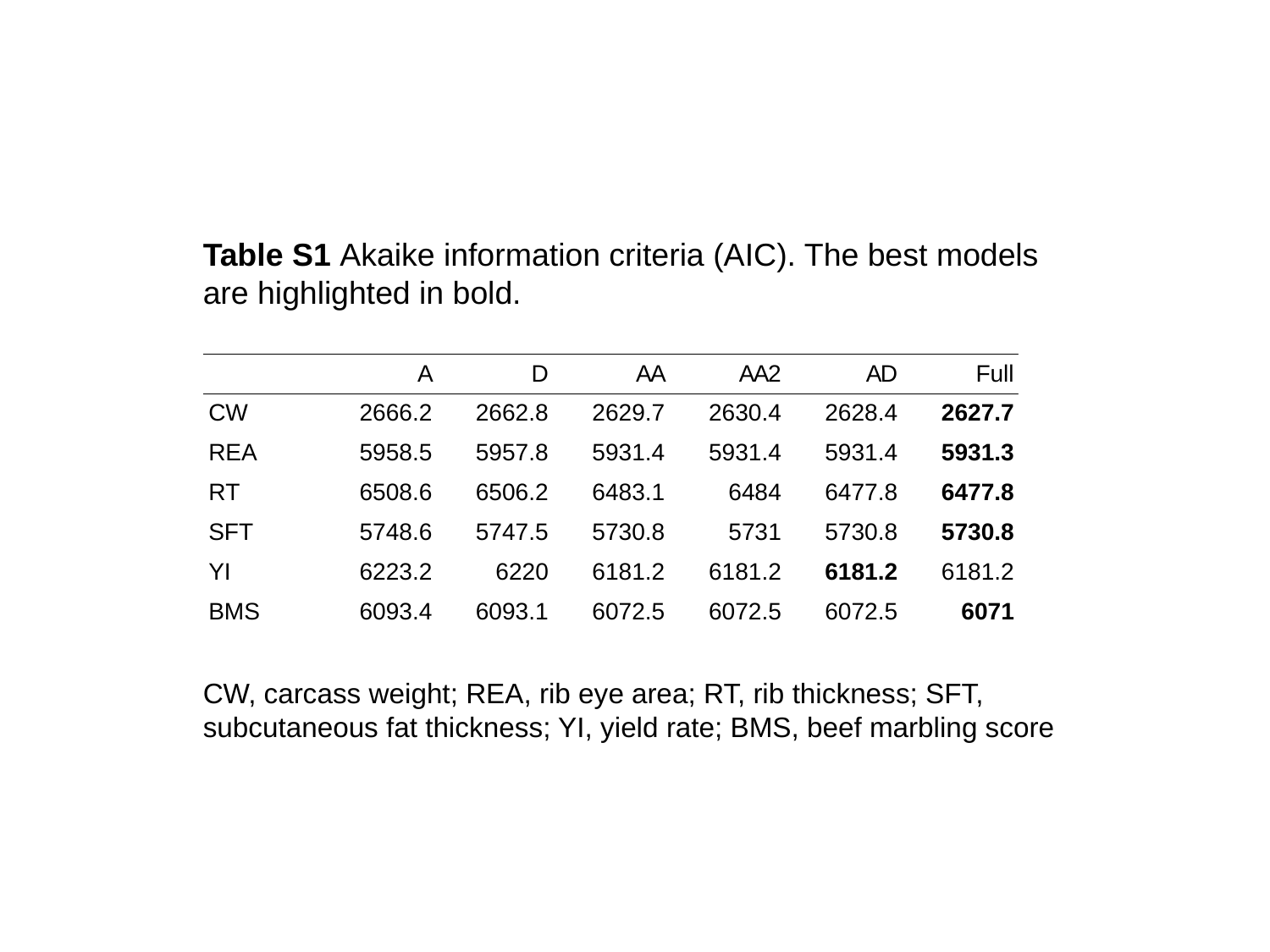

Table S1 Akaike information criteria (AIC). The best models are highlighted in bold.
CW, carcass weight; REA, rib eye area; RT, rib thickness; SFT, subcutaneous fat thickness; YI, yield rate; BMS, beef marbling score

## Slide 4
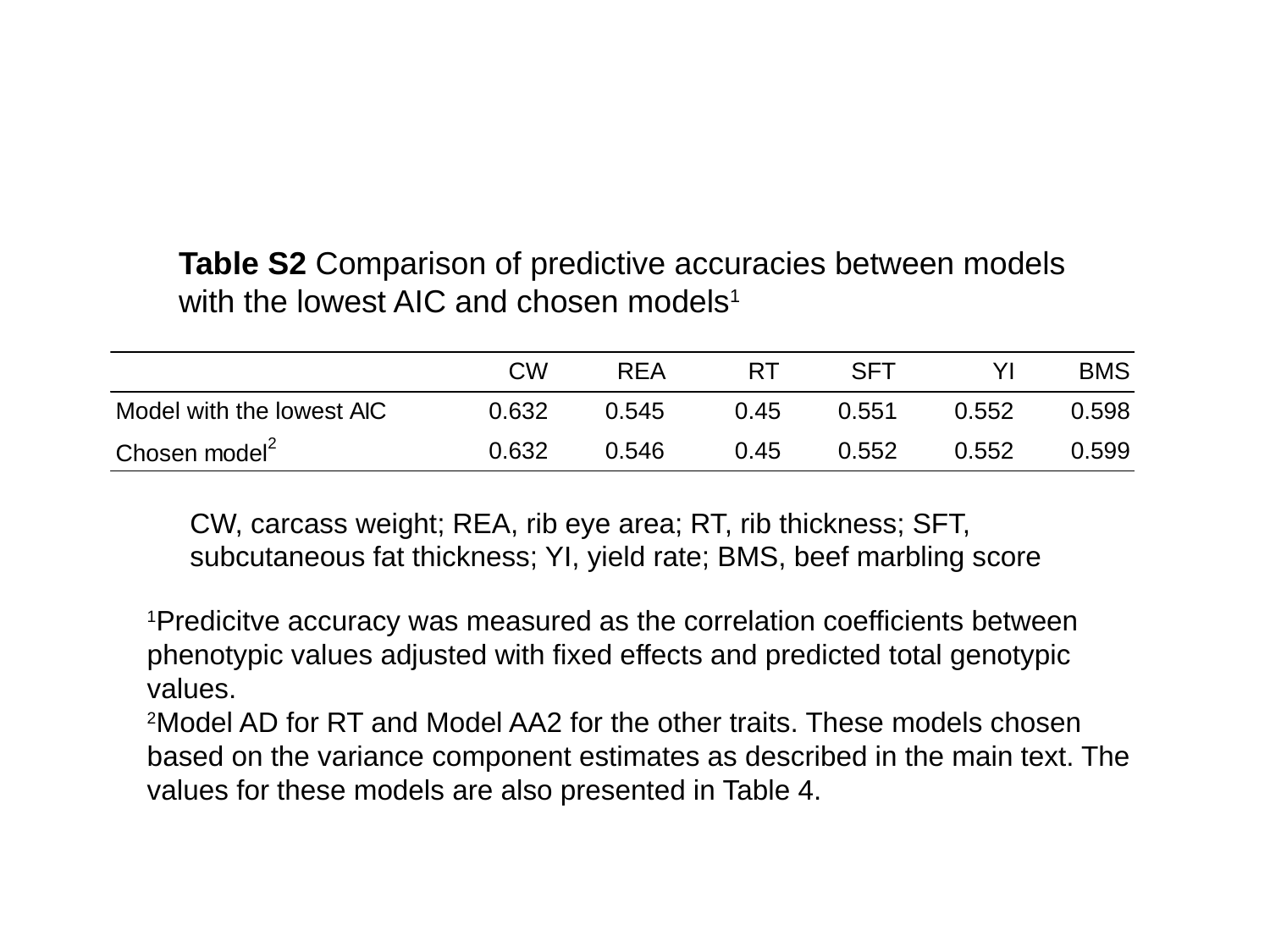

Table S2 Comparison of predictive accuracies between models with the lowest AIC and chosen models1
CW, carcass weight; REA, rib eye area; RT, rib thickness; SFT, subcutaneous fat thickness; YI, yield rate; BMS, beef marbling score
1Predicitve accuracy was measured as the correlation coefficients between phenotypic values adjusted with fixed effects and predicted total genotypic values.
2Model AD for RT and Model AA2 for the other traits. These models chosen based on the variance component estimates as described in the main text. The values for these models are also presented in Table 4.

## Slide 5
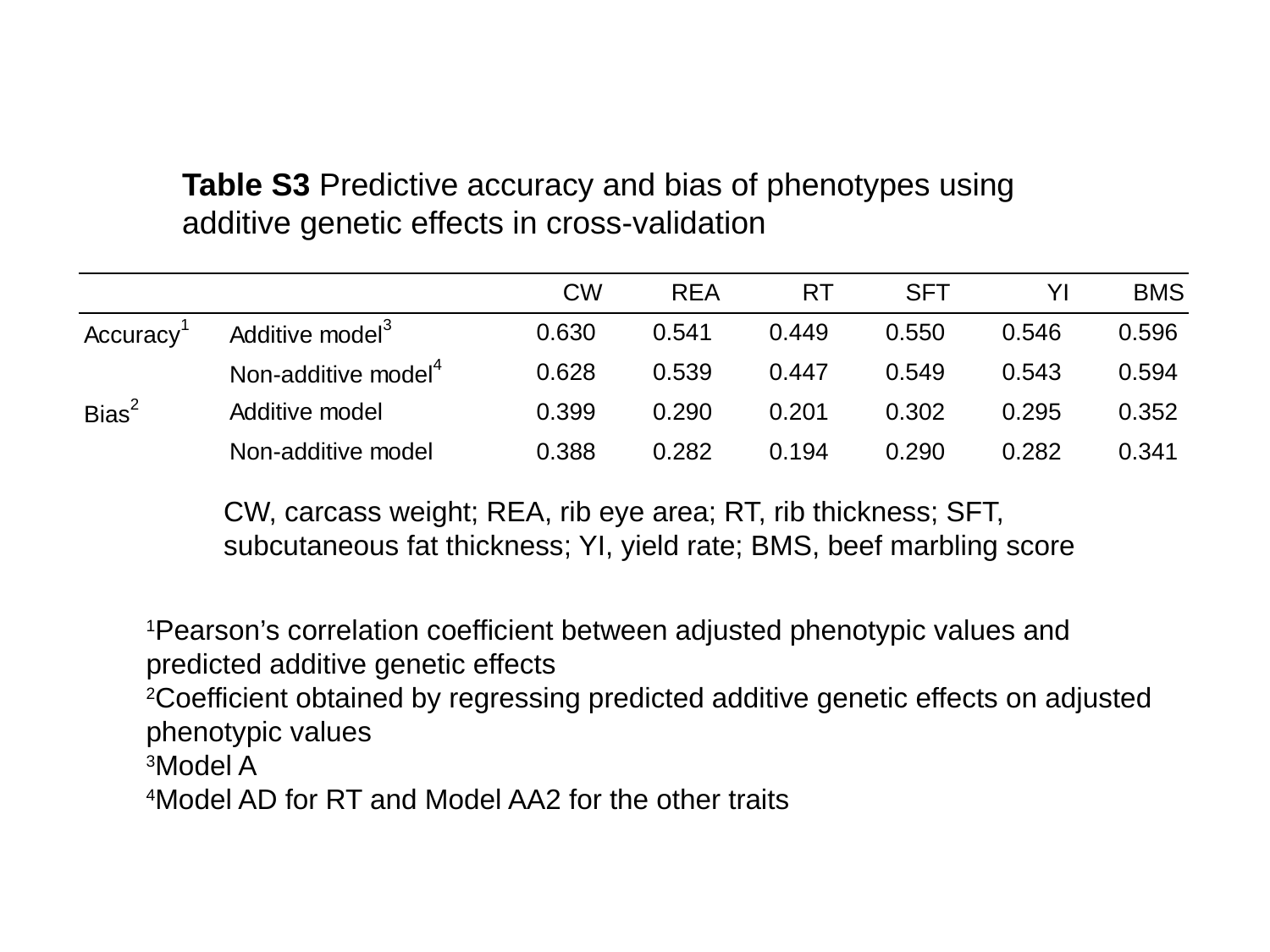

Table S3 Predictive accuracy and bias of phenotypes using additive genetic effects in cross-validation
CW, carcass weight; REA, rib eye area; RT, rib thickness; SFT, subcutaneous fat thickness; YI, yield rate; BMS, beef marbling score
1Pearson’s correlation coefficient between adjusted phenotypic values and predicted additive genetic effects
2Coefficient obtained by regressing predicted additive genetic effects on adjusted phenotypic values
3Model A
4Model AD for RT and Model AA2 for the other traits
